# Myocardial Work in Middle-Aged Adults with Overweight and Obesity: Associations with Sex and Central Arterial Stiffness

**DOI:** 10.3390/jcm12175676

**Published:** 2023-08-31

**Authors:** Katrine Tryti Lindseth, Eva Gerdts, Helga Midtbø, Nadia Pristaj, Dana Cramariuc, Eigir Einarsen

**Affiliations:** 1 Center for Research on Cardiac Disease in Women, Department of Clinical Science, University of Bergen, 5020 Bergen, Norway; trytikatrine@gmail.com (K.T.L.);; 2 Department of Heart Disease, Haukeland University Hospital, 5021 Bergen, Norway; helga.bergljot.midtbo@helse-bergen.no (H.M.); dana.cramariuc@helse-bergen.no (D.C.); 3 Department of Medicine, Nordland Hospital Trust, Vesterålen Hospital, 8450 Stokmarknes, Norway; eigir.einarsen@gmail.com

**Keywords:** global myocardial work, arterial function, sex, obesity

## Abstract

We explored global myocardial work index (GWI), a novel measure of myocardial function that integrates left ventricular (LV) hemodynamic load, in relation to sex and increased body mass index (BMI). We used data from 467 individuals (61% women, average age 47 ± 9 years and BMI 31.2 kg/m^2^) without known cardiac disease. Central arterial function was analysed by applanation tonometry. GWI was calculated from global longitudinal strain (GLS) and post-echocardiography supine blood pressure (BP). Covariables of GWI were identified in linear regression analyses. Women had higher BMI, aortic augmentation pressure (12 ± 7 vs. 8 ± 6 mmHg), LV GLS (20.0 ± 2.8 vs. 18.8 ± 2.8%), and GWI (2126 ± 385 vs. 2047 ± 389 mmHg%) than men (all *p* < 0.05). In univariable analyses, higher GWI was associated with female sex, higher age, systolic BP, LV wall stress, LV ejection fraction, left atrial size, LV ejection time, and with lower waist circumference (all *p* < 0.05). In multivariable analysis, adjusting for these correlates, female sex remained independently associated with higher GWI (*β* = 0.13, *p* = 0.007). After additional adjustment for aortic augmentation pressure or central pulse pressure, this association became non-significant. In conclusion, the higher GWI in women compared to men was mainly explained by increased LV workload due to higher aortic augmentation pressure in women.

## 1. Introduction

Obesity is a major risk factor for hypertension and strongly associated with development of organ damage like arterial stiffness, left ventricular (LV) hypertrophy, and left atrial dilatation, with higher prevalence rates among women [1,2,3]. Previous studies involving subjects with increased body mass index (BMI) have demonstrated that although LV systolic function measured by ejection fraction often is normal, LV myocardial function assessed by global longitudinal strain (GLS) is frequently reduced [4]. At the same time, women tend to have better GLS compared to men [5,6,7], consistent with higher LV ejection fraction and midwall shortening in women [8]. GLS is a robust marker of early LV systolic dysfunction [9], but it is negatively influenced by higher blood pressure (BP), central arterial stiffening, and backward wave reflections, reflecting its afterload dependency [10,11].

Recently, a novel method to quantify LV myocardial work by pressure-strain loops was introduced [12]. The method is based upon combining speckle tracking strain imaging and a non-invasive estimate of LV pressure. Myocardial work estimated by this method has been shown to be closely related to invasively estimated work and metabolism [12]. In the population-based Copenhagen City cohort study, LV myocardial work indices differed by sex and changed with age in a sex-dependent manner [13]. However, the sex differences were small, and abnormal myocardial work indices were particularly associated with cardiovascular risk factors, markers of increased afterload, and abnormal LV geometry [13]. In the Characteristics and Course of Heart Failure STAges A/B and Determinants of Progression cohort study, obesity was associated with lower and hypertension with higher global work index (GWI), and these associations were stronger in women [14]. However, none of these previous studies adjusted for the documented sex differences in arterial function [15,16], which may contribute significantly to LV load. Thus, the aim of the present study was to assess the impact of central aortic function on LV myocardial work indices in middle-aged women and men with increased BMI.

## 2. Materials and Methods

### 2.1. Study Population

The present analysis is a substudy of the cross-sectional FAT-associated CardiOvasculaR dysfunction (FATCOR) study that was conducted in Bergen, Norway, from 2009 to 2017 as a collaboration between the Department of Heart Disease, Haukeland University Hospital, and a general practitioner center that focuses on management of obesity. Details regarding the study protocol have been published [17]. Briefly, a total of 620 women and men between 30 and 65 years with a BMI greater than 27 kg/m^2^ were included. Exclusion criteria were previous cardiovascular disease, gastrointestinal disorders, severe psychiatric illness, or inability to understand Norwegian language. For the present study, we included 595 participants with echocardiograms taken using a GE Vivid E9 machine (GE Vingmed Ultrasound, Horten, Norway). Among these participants, 2 withdraw consent, 2 were excluded due to missing BP measurements, 51 due to incomplete echocardiographic data, 49 due to inadequate echocardiographic image quality for strain analysis, and 24 due to missing segmental strain values in ≥2 LV segments. This resulted in a total of 467 (78%) participants eligible for the present analysis.

### 2.2. Cardiovascular Risk Factor Assessment

All participants underwent a standardized clinical examination at the general practitioner center. Additionally, participants completed a standardized questionnaire on self-reported health, including use of any medication. The provided information was quality assured by a study nurse. Waist circumference was measured using a nonflexible measuring tape as recommended by the World Health Organization. BMI was calculated from weight in kilograms divided by the square of body height in meters (kg/m^2^). Obesity was defined as a BMI ≥ 30.0 kg/m^2^.

BP was measured using an Omron M4 sphygmomanometer (Omron Healthcare Co. Ltd., Hoofddorp, The Netherlands) with an appropriately sized cuff. BP was measured in triplicates with 1 min intervals after 5 min of initial rest in the seated position [18]. Clinic BP was calculated as the average of the last two BP measurements. BP was also measured at completion of the echocardiographic examination, with the participant supine on the examination table with dimmed lights. Ambulatory 24-hour BP was recorded using a Diasys Integra II apparatus (Novacor, Cedex, France) on the non-dominant arm. Measurements were performed every 20 min during daytime and every 30 min at night. Hypertension was defined as use of antihypertensive medication or an elevated 24-hour ambulatory BP (≥130 mmHg systolic or ≥80 mmHg diastolic) in the individual participant.

A standardized 2-hour oral glucose tolerance test was performed in all participants without known diabetes mellitus (DM). DM was defined as a previous history of diabetes, glycated haemoglobin A_1c_ (HbA_1c_) ≥ 6.5%, fasting plasma glucose ≥ 7 mmol/L, or a 2-hour plasma glucose ≥ 11.1 mmol/L during an oral glucose tolerance test.

### 2.3. Central Arterial Stiffness and Pulse Wave Analysis

Central arterial stiffness and aortic hemodynamic parameters were assessed by applanation tonometry using a SphygmoCor apparatus (AtCor Medical, Sydney, Australia). Carotid-femoral pulse wave velocity (PWV) was calculated as previously reported [17]. Central aortic parameters, including central pulse pressure (cPP), augmentation pressure (AP), and augmentation index (AIx), were derived from pulse wave analysis of the right carotid artery pressure waveform. AP was defined as the difference between the second and first systolic pressure peaks of the central pressure waveform. AIx was calculated as AP/cPP and expressed as a percentage.

### 2.4. Conventional Echocardiography

Transthoracic two-dimensional echocardiography was performed following a standardized protocol in all participants. Echocardiographic images were analysed off-line in the Echocardiography Core Laboratory at the University of Bergen, Norway, using Tomtec Image Arena Software version 4.6 (Tomtec Imaging Systems GmbH, Unterschleissheim, Germany).

Quantitative echocardiography was assessed following joint guidelines from the American Society of Echocardiography and the European Association of Cardiovascular Imaging [8]. LV hypertrophy was defined as LV mass index > 47.0 g/m^2.7^ in women and >50.0 g/m^2.7^ in men, respectively [18,19]. Left atrial volumes and left ventricular ejection fraction were assessed by the biplane Simpson’s method. Stroke volume was calculated by the Doppler method. Left atrial volume was indexed for height^2^ and stroke volume was indexed to height at the allometric power of 2.04 [20]. Meridional LV wall stress was estimated using a validated equation [21]. Systolic ejection time was measured as the time interval in milliseconds from aortic valve opening to closure [22].

### 2.5. Myocardial Work Analysis

**Figure 1:**
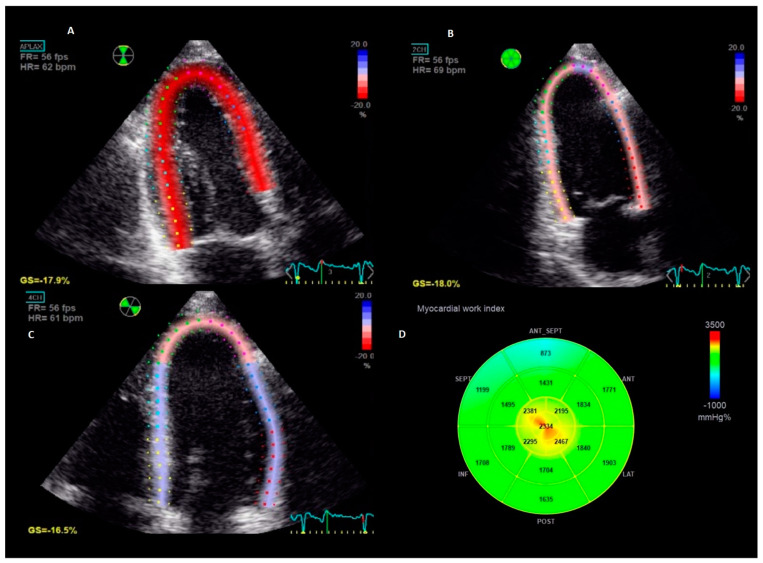
Measurement of myocardial work in an individual patient. The figure represents the longitudinal strain imaging from the standard apical-three-chamber (Panel (**A**)), two-chamber (Panel (**B**)) and four-chamber (Panel (**C**)) views and the corresponding bull`s eye of global work index calculated from the area within the pressure-strain loop (Panel (**D**)).

Two-dimensional speckle tracking analysis was performed offline on a dedicated workstation equipped with EchoPac BT 202 (GE Vingmed Ultrasound, Horten, Norway). Apical two-, three-, and four-chamber views with an optimized frame rate were analysed using the Automated Function Imaging application. GLS was calculated as the average peak systolic negative longitudinal shortening of the 18 LV segments. Since GLS is expressed as a negative percentage, we report the absolute values |x| for a simpler interpretation. Timing of mitral and aortic valve opening and closure were determined using pulsed-wave Doppler signals. The supine brachial BP measured at the end of the echocardiographic examination was used in the calculation of LV myocardial work. By synchronizing longitudinal strain, BP, and timing of valvular events, non-invasive pressure-strain loops (PSL) were provided (Figure 1). The area within the PSL corresponds to a global myocardial work index (GWI), calculated from mitral valve closure to mitral valve opening. In addition, other indices of myocardial work such as global constructive work (GCW, the work performed during LV shortening in systole and LV lengthening during isovolumic relaxation), global wasted work (GWW, myocardial work not contributing to LV ejection due to lengthening in systole and LV shortening during isovolumic relaxation), and global work efficiency (GWE, constructive work divided by the sum of constructive and wasted work) were calculated [23].

### 2.6. Statistical Analysis

Data management and statistical analyses were performed using IBM SPSS version 26 (IBM Corporation, Armonk, NY, USA) and R version 1.2.5042 (R Foundation for Statistical Computing, Vienna, Austria) with the package “cocor”. The study population was grouped by sex. Data are presented as mean ± standard deviation for normally distributed variables, median with interquartile range (25–75 percentile) for non-normally distributed variables, and as percentages for categorical variables. Group comparisons were performed using Student’s *t*-test for continuous variables, Mann–Whitney U test for non-normally distributed variables, and Pearson’s chi-square test for categorial variables. For continuous variables, normality was checked by Q-Q plots and visual assessment of histograms prior to analyses. Homogeneity of variance was assessed using Levene’s test. The Spearman’s rank-order correlation (ρ) was used to assess univariable associations between GWI and covariables. The Spearman correlation coefficients between GWI and clinic systolic and central systolic BP, respectively, were normalized by Fisher’s z-transformation and compared by z-statistics. Multivariable linear regression analyses with collinearity tools were used to identify independent covariables of GWI. The multivariable models were constructed as follows: In Model 1, we included all correlates among clinic and echocardiographic variables with a *p* value < 0.1 in univariable analyses using a stepwise procedure. In Model 2, we added AP, the central aortic variable that had the highest correlation coefficient with GWI and was statistically different between sexes. In a subsequent model, AP was replaced by cPP. The AP is known to be significantly influenced by body height, sex, and heart rate. The relative importance of these covariables to GWI was determined with analysis of variance (ANOVA) using the F statistics and the Akaike information criterion (AIC). In sex specific analyses, significant univariable correlations by sex were added to a primary multivariable model by a similar procedure as mentioned above. Secondary multivariable models for each central aortic parameter, adjusted for otherwise significant correlates of GWI by sex, were constructed using an enter procedure. Results are presented as standardized β coefficients and *p* values. Interobserver reproducibility for GLS was calculated by intraclass correlation coefficient after reanalysis of 26 randomly selected participants. A two tailed *p* value < 0.05 was considered statistically significant in all analyses.

## 3. Results

### 3.1. Patient Characteristics

The present analysis included a total of 467 participants from the FATCOR study (61% women). The average age was 47 ± 9 years, and the median BMI was 31.2 (28.9–33.8) kg/m^2^. Women had higher BMI, AP, and AIx, but lower waist circumference, clinic and 24-hour systolic BP and PWV, and lower prevalence of hypertension (all *p* < 0.05) (Table 1). cPP and the prevalence of obesity and diabetes did not differ between sexes (Table 1).

### 3.2. Echocardiographic Findings

**Figure 2:**
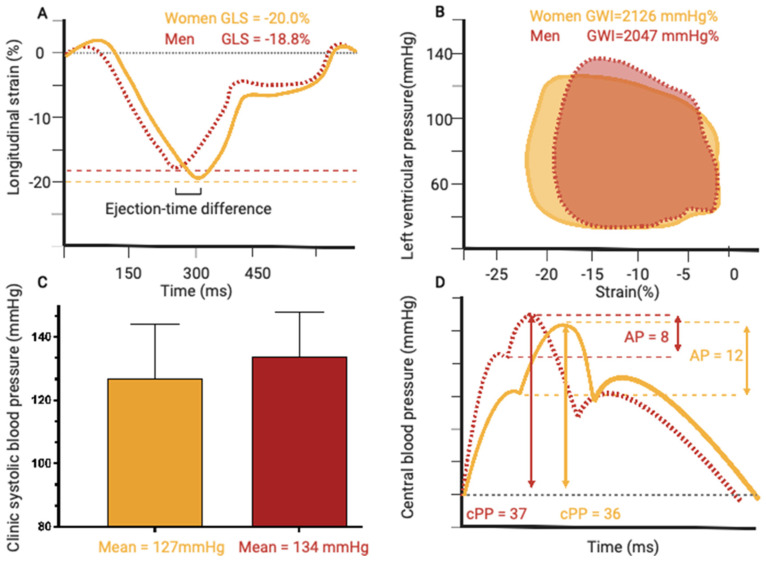
Graphical representation of mean differences between women and men in; (**A**) global longitudinal strain, (**B**) clinic systolic blood pressure, (**C**) pressure-strain loops and global work index, (**D**) central arterial waveforms.

LV hypertrophy and LV ejection fraction did not differ between sexes, while GLS was higher in women (*p* < 0.001) (Table 2) (Figure 2). Among measures of myocardial work, GWI was higher in women (*p* = 0.031), while GCW, GWW, and GWE did not differ between sexes. Interobserver reproducibility for GLS was excellent, with an intraclass correlation coefficient of 0.96 (95% CI, 0.95–0.98).

### 3.3. Covariables of Global Work Index

The univariable correlates with GWI are listed in Table 3. In univariable analysis, GWI showed a strong correlation with clinic systolic BP (ρ = 0.29, *p*-value < 0.001), post-echocardiography systolic BP (ρ = 0.51, *p*-value < 0.001), and central systolic BP (ρ = 0.37, *p*-value < 0.001). In Z-statistics, central systolic BP had a borderline stronger correlation with GWI than clinic systolic BP (z = −1.98, *p* value = 0.047). Due to collinearity between the supine post-echocardiography systolic BP and GWI, and between clinic and central systolic BP, only clinic systolic BP was included in the multivariable regression models to avoid collinearity and overfitting of the regression model. In multivariable analysis, including all significant univariable variables, except central aortic parameters, female sex was independently associated with higher GWI (Table 3, Model 1). When adding AP to the model, higher AP was significantly associated with higher GWI (β = 0.12, *p* = 0.024) while the association between GWI and female sex became non-significant (Table 3, Model 2). Due to collinearity, AP and cPP were not included in the same model. When AP was replaced by cPP, higher cPP was associated with higher GWI (β = 0.19, *p* < 0.001), and female sex remained non-significant. The relative contributions to GWI of known clinical correlates of AP are presented in Supplemental Material Figure S1. This analysis revealed that GWI was predominantly determined by AP, while variables known to influence AP, such as height, sex, and heart rate, had little impact. Substituting AP with cPP in the model gave similar results.

### 3.4. Sex-Specific Analyses

Higher GWI was associated with increased arterial stiffness in both sexes in univariable analyses (Table 4). The correlation between GWI and LV ejection time was significant only in women (ρ = 0.28, *p* < 0.01 vs. ρ = 0.13, *p* = 0.08 in men). In the primary multivariable analysis, GWI correlated with higher systolic BP, wall stress, LV ejection fraction, left atrial volume index, and LV systolic ejection time in women (all *p* < 0.01). Among men, higher GWI correlated with lower waist circumference, higher systolic BP, wall stress, ejection fraction, and left atrial volume index (all *p* < 0.011). In a series of secondary sex-specific models, each including one central aortic parameter and adjusting for the covariables identified in the primary model, cPP remained the most important covariable of increased GWI in both sexes (Table 4). AP remained significantly associated with higher GWI in women, but not in men (Table 4). There were no significant associations between higher GWI and AIx or PWV in either sex.

## 4. Discussion

The present study provides new insight into the interaction of central aortic function with LV myocardial work in women and men with increased BMI. Our findings suggest that higher GWI among women with increased BMI may reflect a higher arterial load. Importantly, the association between AP and GWI was independent of heart rate and body height, factors which are known to differ between sexes and to significantly influence AP [24]. Despite a lower PWV in women, AP was higher and cPP comparable between sexes. This is in concordance with a previous study showing that cPP is mainly determined by wave reflections in the proximal aorta [25]. Among individuals younger than 50 years, as in the present study cohort, AIx, similar to AP, is a more sensitive marker of arterial aging and risk [16].

It should be noted that cPP is also influenced by LV ejection time [26]. A longer ejection time implies a lower volume ejected at the time of the first peak of the pressure waveform, which is a major component of the cPP [26]. We observed that women had a significantly longer LV ejection time compared to men, in line with previous reports [27,28]. Longer ejection time also correlated with a higher GWI, and more strongly in women compared to men. Arterial stiffness may contribute to a prolonged ejection because early wave reflections may independently increase the duration of systole [29]. The Framingham Heart Study reported that the longer ejection time in women compared to men could partly account for the observed sex differences in AP [30]. However, in the present study, the associations between LV ejection time, AP, and GWI were relatively independent of each other.

Previous results from the FATCOR study showed that women have higher prevalence of LV atrial dilation, which was associated with higher brachial PP [1]. In the Framingham Heart Study, the more pronounced increase in PP with aging in women was strongly associated with proximal aortic stiffening [31]. The interplay between higher PP, higher LV systolic function, and more subclinical cardiac disease in women could be reflected by a higher GWI, as a measure of increased LV metabolic demand [12]. Peterson et al. showed in a study combining echocardiography and positron emission tomography that women with obesity had significantly higher cardiac work, better LV systolic function, and increased myocardial oxygen consumption compared to men with obesity [32]. As demonstrated by the present study, sex differences in central arterial function may contribute to the higher GWI in women, adding to previous studies assessing clinic BP and obesity [13,14]. Furthermore, wave reflections during mid-to-late systole, as represented by AP, increase peak myocardial wall stress which is a primary determinant of myocardial oxygen consumption [11], and also an independent covariate of higher GWI in the present study.

In line with previous findings, GWI was strongly associated with higher systolic BP [14,33]. However, the correlation between GWI and clinic systolic BP in the present study was weaker compared to findings from the NORRE Study [33]. The differential findings may be related to that NORRE used clinic BP for calculation of GWI, while the supine post-echocardiography BP was used in the present study, in line with the original concept [23]. Even so, with higher systolic BP, the LV shifts to a higher energy level by an increase in inotropy to counteract the higher afterload during contraction [34].

### Study Limitations

The present study has some limitations. Firstly, due to the cross-sectional study design, cause–effect relationships could not be explored. Use of antihypertensive treatment was not associated with higher GWI in multivariable analysis, probably reflecting that a large proportion of the patients were diagnosed with de-novo hypertension during the study based on 24 h BP recording. The cross-sectional study design precludes analysis of antihypertensive drug treatment effects on GWI. Furthermore, the impact of sex differences in longitudinal changes BP and arterial stiffness over the life course on LV myocardial work could not be assessed [35,36]. The trajectory of arterial stiffness progression during midlife is accentuated by menopause transition among women. However, our data collection did not include measurement of follicle-stimulating hormone levels or information regarding the time since last menstruation. We were therefore unable to assess the influence of menopause and subsequent accelerated arterial aging on our findings. However, patients’ age was included in the multivariable models. It should be noted that our cohort was middle-aged individuals with increased BMI, but without known cardiac disease. Since we did not include normal weight individuals, it remains to be demonstrated whether our results are valid also when BMI is normal. Lastly, echocardiographic evaluations of GLS and GWI could be affected by suboptimal image quality and orientation, although we carefully excluded participants with insufficient image quality.

## 5. Conclusions

Women with increased BMI have higher GWI compared to men. In the present cohort, the sex difference in GWI was mainly explained by higher AP in women.

## Figures and Tables

**Table 1:** Characteristics of the total study population and groups of women and men.

Variables	Total Study Population (*n* = 467)	Women (*n* = 284)	Men (*n* = 183)	*p* Value
Age (years)	47 ± 9	48 ± 9	47 ± 9	0.373
Height (cm)	173 ± 9	167 ± 6	180 ± 7	<0.001
Weight (kg)	95 ± 15	90 ± 14	102 ± 13	<0.001
Waist circumference (cm)	107 ± 11	106 ± 12	110 ± 10	<0.001
BMI (kg/m^2^)	31.2 (28.9–33.8)	31.4 (29.0–34.3)	31.0 (28.7–33.2)	0.046
Obesity (%)	62	63	61	0.606
Clinic systolic BP (mmHg)	129 ± 17	127 ± 17	134 ± 14	<0.001
Clinic diastolic BP (mmHg)	82 ± 9	80 ± 9	84 ± 10	<0.001
Clinic heart rate (beats/min)	67 ± 10	69 ± 10	65 ± 11	<0.001
Echocardiography systolic BP	131 ± 16	129 ± 16	136 ± 16	<0.001
24 h systolic BP (mmHg)	121 ± 13	119 ± 12	125 ± 12	<0.001
24 h diastolic BP (mmHg)	79 ± 8	78 ± 8	82 ± 8	<0.001
24 h heart rate (beats/min)	75 ± 9	76 ± 9	73 ± 9	0.001
Hypertension (%)	63	60	69	0.047
Hypertension treatment (%)	22	23	22	0.790
HbA_1c_ (%)	5.6 ± 0.5	5.6 ± 0.4	5.6 ± 0.6	0.898
Diabetes mellitus (%)	8	9	7	0.311
Total cholesterol (mmol/L)	5.4 ± 1.0	5.5 ± 1.0	5.3 ± 1.0	0.092
Triglycerides (mmol/L)	1.3 (0.9–1.7)	1.2 (0.9–1.7)	1.4 (1.0–2.0)	<0.001
LDL cholesterol (mmol/L)	3.6 ± 0.9	3.6 ± 0.9	3.7 ± 1.0	0.674
HDL cholesterol (mmol/L)	1.3 ± 0.3	1.4 ± 0.3	1.1 ± 0.3	<0.001
eGFR (mL/min/1.73 m^2^)	96 ± 13	96 ± 14	98 ± 12	0.080
Pulse wave velocity (m/s)	7.2 (6.4–8.4)	7.0 (6.3–8)	7.6 (6.7–8.6)	0.001
Central systolic BP (mmHg)	116 ± 15	115 ± 16	118 ± 13	0.028
Central pulse pressure (mmHg)	36 ± 10	36 ± 11	37 ± 9	0.212
Augmentation pressure (mmHg)	11 ± 7	12 ± 7	8 ± 6	<0.001
Augmentation index (%)	28 ± 14	33 ± 11	21 ± 14	<0.001

BMI, body mass index; BP, blood pressure; LDL, low density lipoprotein; HDL, high density lipoprotein; HbA_1c_, haemoglobin A_1c_; eGFR, estimated glomerular filtration rate.

**Table 2:** Echocardiographic findings in the total study population and in groups of women and men.

Variables	Total Study Population (*n* = 467)	Women (*n* = 284)	Men (*n* = 183)	*p* Value
Left ventricle				
LV end-diastolic diameter (mm)	50 ± 5	48 ± 4	51 ± 5	<0.001
LV end-systolic diameter (mm)	33 ± 4	32 ± 4	34 ± 4	<0.001
Septal wall thickness (mm)	11 ± 2	10 ± 2	12 ± 2	<0.001
Posterior wall thickness (mm)	8 ± 2	8 ± 1	9 ± 2	<0.001
Relative wall thickness	0.34 ± 0.08	0.33 ± 0.07	0.36 ± 0.08	<0.001
LV mass index (g/m^2.7^)	38 (33–43)	37 (32–37)	40 (35–47)	<0.001
LV hypertrophy (%)	14	12	18	0.052
Meridional wall stress (dyne/cm^2^)	146 ± 27	145 ± 27	148 ± 27	0.239
Systolic function				
Ejection fraction (%)	62 ± 5	62 ± 5	62 ± 5	0.293
Stroke volume index (mL/m^2.04^)	32 ± 7	32 ± 7	32 ± 7	0.234
Global longitudinal strain (%)	19.6 ± 2.9	20.0 ± 2.8	18.8 ± 2.8	<0.001
Systolic ejection time (ms)	306 ± 28	311 ± 28	297 ± 27	<0.001
Diastolic function				
Filling pressure (E/e′)	9.3 ± 2.7	9.5 ± 2.7	9.0 ± 2.8	0.056
Average e′ (cm/s)	9.7 ± 2.2	9.9 ± 2.3	9.3 ± 2.1	0.008
Peak tricuspid regurgitation (m/s)	2.19 ± 0.50	2.21 ± 0.50	2.15 ± 0.51	0.362
Left atrial volume index (mL/m^2^)	20.2 ± 5.7	20.3 ± 5.6	19.9 ± 5.9	0.376
Isovolumetric relaxation time (ms)	91 ± 22	89 ± 14	94 ± 30	0.019
Myocardial work				
Global work index (mmHg%)	2095 ± 388	2126 ± 385	2047 ± 389	0.031
Global constructive work (mmHg%)	2333 ± 407	2333 ± 407	2333 ± 409	0.995
Global wasted work (mmHg%)	75 (49–115)	73 (49–106)	77 (50–126)	0.186
Global work efficiency (%)	96 (95–97)	96 (94–97)	96 (94–97)	0.138

LV, left ventricular.

**Table 3:** Univariable Spearman correlations and multivariable linear regression analyses of covariables of GWI.

	Univariable		Multivariable Model 1		Multivariable Model 2	
	Spearman Correlation Coefficient	*p* Value	Standardized *β* Coefficient	*p* Value	Standardized *β* Coefficient	*p* Value
Female sex	0.13	0.007	0.15	0.001	0.04	0.544
Age (years)	0.25	<0.001	0.12	0.009	0.07	0.154
Clinic systolic BP (mmHg)	0.29	<0.001	0.23	<0.001	0.19	<0.001
Heart rate (beats/min)	−0.09	0.044	−0.12	0.016	−0.09	0.062
Meridional wall stress (dyne/cm^2^)	0.22	<0.001	0.21	<0.001	0.20	<0.001
Ejection fraction (%)	0.20	<0.001	0.24	<0.001	0.23	<0.001
Systolic ejection time (ms)	0.24	<0.001	0.13	0.005	0.12	0.013
Left atrial volume index (mL/m^2^)	0.22	<0.001	0.15	0.001	0.13	0.001
LV mass index (g/m^2.7^)	0.12	0.009				
Hypertensive treatment (yes/no)	0.14	0.003				
Filling pressure (E/e′)	0.18	<0.001				
HbA_1c_ (%)	0.11	0.019				
Waist circumference (cm)	−0.13	0.006				
Height (cm)	−0.17	<0.001			−0.08	0.181
Augmentation pressure (mmHg)	0.33	<0.001			0.12	0.024
Central pulse pressure (mmHg)	0.35	<0.001				
Augmentation index (%)	0.21	<0.001				
Pulse wave velocity (m/s)	0.16	0.001				
BMI (kg/m^2^)	−0.03	0.532				

BP, blood pressure; LV, left ventricular; BMI, body mass index.

**Table 4:** Sex-specific univariable Spearman correlations and multivariable linear regression analyses of the association of individual measures of arterial wave reflection and stiffness with GWI.

Variables	Women				Men			
	Univariable		Multivariable *		Univariable		Multivariable †	
	Spearman Correlation Coefficient	*p* Value	Standardized *β* Coefficient	*p* Value	Standardized *β* Coefficient	*p* Value	Standardized *β* Coefficient	*p* Value
cPP	0.41	<0.001	0.24	<0.001	0.33	<0.001	0.20	0.017
AP	0.34	<0.001	0.19	0.001	0.23	0.002	0.11	0.174
Aix	0.19	0.001	0.08	0.151	0.15	0.038	0.05	0.511
PWV	0.15	0.011	0.01	0.848	0.21	0.004	0.06	0.443

* Adjusted for systolic BP, wall stress, ejection fraction, left atrial volume index, and systolic ejection time; † Adjusted for waist circumference, systolic BP, wall stress, ejection fraction, and left atrial volume index; cPP, central pulse pressure; AP, augmentation pressure; AIx, augmentation index; PWV, pulse wave velocity; BP, blood pressure.

## Data Availability

Data are not available due to ethical restrictions.

## Supplementary Materials

The following supporting information can be downloaded at: https://www.mdpi.com/article/10.3390/jcm12175676/s1, Figure S1: The relative importance of AP, body height, heart rate and sex for GWI by the Akaike information criterion.
