# Supplementary material for: Myocardial Work in Middle-Aged Adults with Overweight and Obesity: Associations with Sex and Central Arterial Stiffness
Source: J Clin Med. 2023 Aug 31;12(17):5676. doi: 10.3390/jcm12175676 (PMC10488455; doi:10.3390/jcm12175676)
Supplement: Supplementary file 1 [file jcm-12-05676-s001.zip › jcm-2546918-supplementary.pdf]

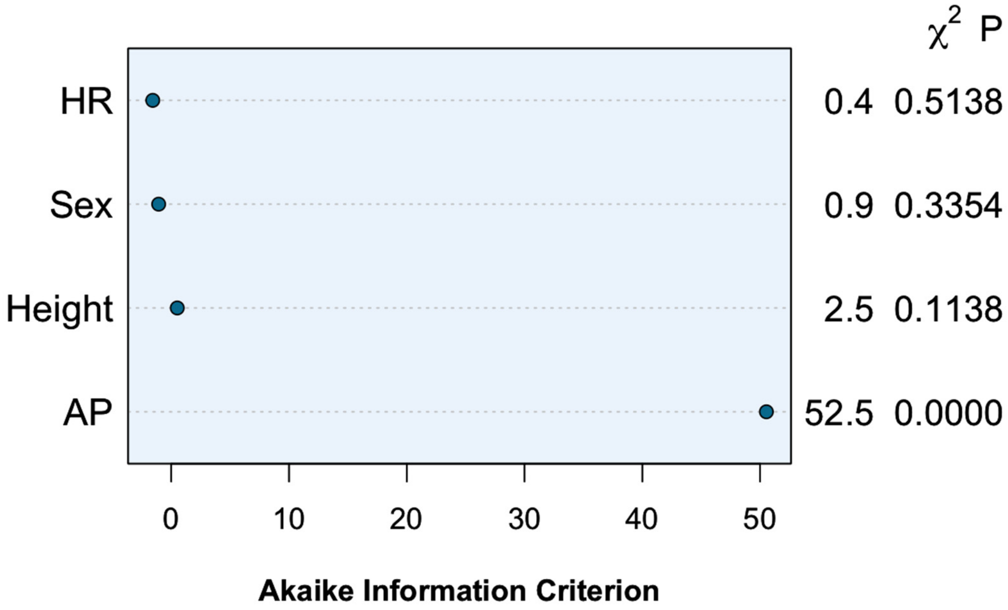


**Figure S1.** Graphical presentation of the relative importance of covariables of GWI. HR, heart rate; AP, augmentation pressure.
